# Design and effectiveness of an online group logotherapy intervention on the mental health of Iranian international students in European countries during the COVID-19 pandemic

**DOI:** 10.3389/fpsyt.2024.1323774

**Published:** 2024-02-21

**Authors:** Shirin Rahgozar, Lydia Giménez-Llort

**Affiliations:** ^1^Department of Psychiatry and Forensic Medicine, School of Medicine, Universitat Autònoma de Barcelona, Barcelona, Spain; ^2^Institut de Neurociències, Universitat Autònoma de Barcelona, Barcelona, Spain

**Keywords:** logotherapy, group psychotherapy, anxiety disorders, depressive disorder, international students, COVID-19 pandemic, migration

## Abstract

**Introduction:**

The secondary impact of the COVID-19 pandemic, leading to widespread psychological challenges, significantly strained international students’ mental health. The present work sought to design and assess the efficacy of an Online Group Logotherapy Protocol, an existential psychology approach developed by Viktor Frankl, to reduce anxiety and depression levels among Iranian international students who were migrants/refugees in different European countries during the pandemic.

**Methods:**

The study recruited 70 students (58 females and 12 males, age range 20–35, 6 EU countries) experiencing moderate levels of anxiety and depression as measured by the Beck Anxiety (BAI) and Depression (BDI) Inventories at pre-test. Half the participants received a short-term closed group intervention comprising 6 online sessions / 90 min of logotherapy. The control group received 6 sessions without specific psychological treatment.

**Results:**

The designed logotherapy sessions consisted of 1. Fundamentals of logotherapy, 2. Existential concerns, 3. Introspection, 4. Self-awareness and growth, 5. Empowering and facing challenges, 6. Meaning of life and conclusions. Five logotherapy techniques were used: Socratic Dialog, Modification of Attitude, Paradoxical Intention, Dereflection, and Logodrama. After the sessions, the post-test MANCOVA analysis showed a more potent effect of logotherapy reducing depression and anxiety than that elicited without intervention. The Eta coefficient suggests that the observed difference explains the effect of logotherapy with a strong power of 89%.

**Conclusion:**

These findings unveil (1) the benefits of online group sessions despite the geographical distance and (2) the relevance of logotherapy effectively reducing depression and anxiety in such complex scenarios where psychological resources and cultural competencies are limited.

## Introduction

The coronavirus pandemic (COVID-19) that broke out in China in late 2019 spread worldwide and ravaged many countries, upending millions of lives. People faced this dangerous and deadly pandemic for months as an unprecedented experience for human beings, and many of them lost loved ones due to the pandemic (1). In addition, a secondary impact of this pandemic was associated with people being forced to change their lifestyles due to severe limitations, shutting down many places, quarantine, isolation, wearing a mask, or confronting severe economic problems. Thus, the unprecedented scenario has profoundly impacted public health and individuals worldwide, presenting unique challenges and stressors (1–4). In the case of international students, where being a foreigner and young were factors of potential vulnerability (5–7), they experienced a significant strain on their mental health when confronting disruptions in their academic and personal lives. Thus, the pandemic has exacerbated feelings of homesickness, loneliness, and depression among this vulnerable group (8, 9). The mental health concerns among international students have been further exacerbated by the challenges posed by the pandemic, including travel restrictions, social isolation, and uncertainty about the future (10). In fact, international students not only confronted the same situation but were also far from their family and friends, enhancing their homesick risk factors and vulnerability to mental health problems. In the case of Iranian international students, their condition of living in different countries as migrants/refugees (11) was added to these factors, highlighting the pressing need for effective mental health interventions tailored to this population. Therefore, in the context of our research project on the use of ‘Logotherapy on Mental Health of Immigrants of the Third Millennium’ (12) and after further literature research on its effectivity, we designed an experimental clinical psychology study to assess the effectiveness of group logotherapy sessions on decreasing levels of anxiety and depression in these international students. For this purpose, a sample of Iranian international students living in European countries who suffered from moderate anxiety and depression during the COVID-19 pandemic was chosen among those who answered our call.

### Logotherapy: an introduction to meaning-centered psychotherapy

Logotherapy, an existential psychotherapy approach developed by Viktor E. Frankl, is founded on the belief that the primary human drive is to find purpose and meaning in all circumstances (13, 14). The crux of logotherapy lies in the relentless pursuit of meaning, even in the face of suffering and adversity (15). The four key tenets of logotherapy are (1) Search for Meaning: Individuals are driven to seek meaning in life, in their actions, experiences, and relationships (13, 14); (2) Freedom of Will: Despite circumstances, individuals possess the freedom to choose their attitude toward situations and how they derive meaning from them (13); (3) Responsibility: logotherapy emphasizes taking responsibility for one’s life, choosing how to respond to situations, and thus ensuring a sense of purpose and meaning (13); (4) Suffering and Meaning: Suffering is seen as an opportunity to find meaning, to transform it into a triumph of the human spirit through the search for purpose (16).

Among various therapeutic approaches, logotherapy, has gained recognition for its efficacy in promoting mental well-being (13, 14, 17). This approach has shown promise in alleviating mental health concerns and providing individuals with a sense of purpose and meaning (18). The core principles of logotherapy resonate with the needs of international students during these challenging times, offering a potential avenue for enhancing their mental health outcomes (17) in these current times restricted by limited clinical psychology resources, and cross-cultural competencies.

This study aimed to develop a protocol adapted to young adult migrants based on existing logotherapy research and investigate its effectiveness in reducing moderate anxiety and depression among Iranian international students during the COVID-19 pandemic. Thus, we first search for existing research protocols of logotherapy in such a clinical psychology field to build the one to be implemented. Then, we hypothesized that the participants receiving the logotherapy intervention when compared to a control group, would demonstrate a substantial impact on their mental health, affirming its potential as a vital therapeutic approach to deal with the uncertain current reality times in their complex cross-cultural clinical scenarios (15, 16). This hypothesis would be assessed by employing rigorous statistical analysis, specifically MANCOVA, which has shown significant effectiveness of logotherapy in similar contexts (16).

## Methods

### Design of the logotherapy intervention

During the inception of this study, the WHO declared COVID-19 as a global pandemic on March 11, 2020 (19). In response, Spain and various other countries implemented strict confinement measures to combat the advancing pandemic (20, 21). Even after a year, confinement measures persisted as an option to curb the virus, causing significant economic, social, and psychological impact (22). Forced confinement led to a range of negative emotions, including frustration, restlessness, sadness, fear, and anger (20, 23). These circumstances have necessitated the reorganization of domestic spaces and increased reliance on virtual systems, adding further stressors (24). The COVID-19 pandemic, like other epidemics, amplifies psychiatric morbidity and induces emotional distress (25).

The professional guidance to Iranian international students during the COVID-19 pandemic to find the meaning of life through interventions such as logotherapy could help them in this process (26). The design of the online group logotherapy intervention was based on two resources: (1) Our previous work (12) where the foundations and applications of logotherapy to improve mental health of immigrant populations were disclosed; (2) The design of the intervention was also based on existing research in the following databases: PubMed, Web of Science, Psychiatry Online, PsycINFO, and MEDLINE, between 2005 and 2021. The terms used to identify relevant studies included ‘logotherapy’, ‘mental health’ and ‘international students’. Only studies that met the following criteria were included in the analysis: (1) Research approach, i.e., quantitative, qualitative, or mixed, is explicitly or implicitly referred; (2) Treatment for mental health symptoms is comprehensively described; (3) The described treatment applied logotherapy principles and techniques; (4) The participants were diagnosed with mental health problem symptoms, such as anxiety, depression, or PTSD. Papers addressing the topic in a general or specific way in other clinical contexts were excluded. During the progress of our project, a second search including 2022 and 2023 to compile emerging literature on the mental status of students during the COVID-19 pandemic was also done.

### Effectiveness of the logotherapy intervention protocol

#### Participants

Iranian international students interested in participating in this study were recruited through the snowball method via unbiased online advertising on social media platforms including Facebook, Twitter, Instagram, and LinkedIn. Advertisements were designed to minimize potential bias, clearly stating the study’s independence and the absence of any affiliation with the researchers. All of them were contacted to verify their profile and to further inform about the study. Rigorous measures, including a one-on-one clinical interview, were employed to verify the absence of disqualifying psychological conditions and treatments, thereby confirming eligibility based on the study’s specific requirements as follows:

**Inclusion Criteria:** Iranian international university students aged between 20 to 35 years, residing in Europe. Individuals experiencing moderate levels of depression and anxiety, as determined by scores on the Beck Anxiety Inventory (BAI) and Beck Depression Inventory (BDI), indicative of the secondary psychological impact of the COVID-19 pandemic.

**Exclusion Criteria:** Current use of any psychiatric medications. Engagement in any other form of psychotherapy at the time of the study. Diagnosis with other psychological disorders, specifically obsessive-compulsive disorder (OCD) or Post-Traumatic Stress Disorder (PTSD), to ensure sample homogeneity.

For this research, we developed a dedicated website where volunteers could find all the necessary information regarding participation requirements. After a brief interview (see below) and informed consent, they were redirected to this website to perform a pre-test screening to confirm suitability to be a participant.

#### Brief interview

The individual clinical interview was a single session conducted for each volunteer who met the initial screening criteria and completed the questionnaires. The interviews were conducted by the primary researcher, who is not only a clinical psychologist but also an experienced psychotherapist. These interviews were integral to the study design, serving to meticulously apply the inclusion and exclusion criteria. During the interviews, the researcher assessed the psychological status of each participant through a standardized set of questions tailored to identify the presence of moderate depression and anxiety related to the COVID-19 pandemic impact.

During the interview, a structured assessment was conducted, which included a review of the volunteers’ medical and psychological histories. This process allowed for a careful consideration of each participant’s suitability for the study, ensuring that their depression and anxiety were indeed attributable to the COVID-19 pandemic, as measured by the BAI and BDI, and not confounded by other factors. Thus, these interviews allowed for the evaluation of other potential psychological conditions that could exclude participants from the study, such as the presence of OCD, PTSD, or current use of psychological medication. This thorough screening process ensured that all participants had a similar baseline related to the specific study parameters, thereby maximizing the internal validity of the research findings. The interview also provided an opportunity to clarify any ambiguities in the questionnaire responses and to establish a baseline for participants’ mental health status.

#### Clinical instruments

The Beck Anxiety Inventory (BAI) and Beck Depression Inventory (BDI) (27, 28) are self-report inventories widely used for their reliability and validity in measuring the severity of anxiety and depression. They were made available on our website platform in both English and the students’ native language, Persian, to ensure comprehension and accuracy in responses. This bilingual approach was designed to accommodate the participants’ language preferences and to enhance the reliability of the self-reported data by allowing students to express their mental health status in the language they are most comfortable with.

The Beck Anxiety Inventory (BAI), a 21-item self-report inventory, is specifically designed to assess the intensity of anxiety in clinical populations. Each item describes a common symptom of anxiety, and respondents are asked to rate how much they have been bothered by that symptom over the past week on a scale of 0 (not at all) to 3 (severely). The BAI has been validated across diverse populations and settings and demonstrates high internal consistency, with Cronbach’s alpha typically ranging from 0.92 to 0.94, and a good test–retest reliability over 1 week with a correlation of 0.75 (28). The Beck Depression Inventory (BDI) consists of 21 items to assess the intensity of depression. It covers affective, cognitive, and somatic symptoms of depression. Like the BAI, respondents rate each item based on their experience over the past 2 weeks. The BDI is known for its high construct and content validity, with Cronbach’s alpha coefficients regularly above 0.86, indicating excellent internal consistency. It also shows high concurrent validity with other measures of depression and a good test–retest reliability coefficient of around 0.93 for 1 week (27).

#### Study design

This study was conducted as a single-blind study (only the therapist and researcher were aware of the participants’ group assignments). The intervention protocol finally consisted (see results section) of 6 group sessions where topics and logotherapy techniques were systematically incorporated, allowing for a comprehensive and targeted approach to addressing the mental health concerns of the participants. The control group received 6 sessions without specific psychological treatment. All participants were under the impression that they were receiving group logotherapy sessions, ensuring consistency in their experiences and minimizing potential biases in their responses.

#### Assessment of efficacy and follow-up on feedback on the online intervention

The individual clinical interviews before the sessions started served as an additional tool to gather important information about the participants’ mental health status and experiences prior to the intervention. The insights gathered from these interviews, along with the pre-test questionnaires and the follow-up on feedback, contribute to a comprehensive evaluation of the intervention’s effectiveness.

To control the effectiveness of the logotherapy online intervention, pre-test and post-test questionnaires were administered to each participant. By comparing the responses before and after the intervention, changes in participants’ mental health could be measured, providing insights into the impact of the logotherapy intervention.

The follow-up on feedback regarding the intervention involved recording the therapy sessions and transcribing the contents of interest. This allowed the researchers to analyze and evaluate the feedback provided by the participants. By reviewing the recorded sessions and analyzing the transcriptions, the researchers gained valuable insights into the participants’ experiences and perceptions of the intervention.

#### Statistical analysis

The results were analyzed using the MANCOVA (Multivariate Analysis of Covariance) test and Student *t*-test comparisons. Assumptions were examined, including canonical correlation, homogeneity of variance–covariance matrices, homogeneity of interactive effects, and homogeneity of regression slopes. All four assumptions were met. Analyses were conducted using IBM SPSS Statistics software (version 26). Statistical significance was considered at *p* < 0.05.

## Results

### Systematic search on logotherapy and mental health

The PRISMA flow chart at four levels was as follows:

*Level 1, Identification:* Following these criteria, the search yielded 430 records (408 after duplicates were removed) as possible analysis sources. After reading the titles of the initially selected articles, 299 papers were included.

*Level 2, Screening:* After reviewing the abstracts, we identified 143 articles for further consideration. Within this group, 39 studies specifically focused on logotherapy and mental health issues. The remaining 104 articles examined the mental health of international students during the COVID-19 pandemic. Notably, there were no studies found that investigated the application of logotherapy to the mental health of international students in the context of the COVID-19 pandemic.

*Level 3, Suitability:* The number of full-text articles considered for eligibility was 112, including 29 on logotherapy and mental health and 83 on international students’ mental health during COVID-19.

*Level 4, Inclusion:* Table 1 summarizes the 26 empirical studies illustrating the effectiveness of logotherapy in various contexts, including managing anxiety and depression (Table 1A, 17 studies) and illuminating the adverse impact of the COVID-19 pandemic on the mental health of international students (Table 1B, 9 articles). Most of these studies demonstrate positive outcomes in depressed patients, aligning with our hypothesis that logotherapy can be an effective psychotherapy for alleviating moderate anxiety and depression resulting from the secondary impact of the COVID-19 pandemic. For instance, Kim and Choi (42), showed in their research that, after participating depressed older adults in logotherapy, they discovered their lives were unique and meaningful. Discovery of the meaning in life helped to reduce their depressive symptoms and to infuse their lives with vitality and confidence. Also, after completing the logotherapy, they wanted to do something meaningful for others. These outcomes have significant implications for preventing depression and improving psychological health in older adults with depressive symptoms, as well as in other countries. Despite few studies on international students, they shed light on the mental health challenges they faced due to the pandemic’s secondary effects. The comprehensive analysis emphasized the urgent need for targeted interventions and support mechanisms to address their unique mental health concerns. In addition, during the progress of the current study, emergent literature supporting the critical status of mental health in international students was found, as summarized in Table 1C.

**Table 1:** Studies on the effectiveness of logotherapy on mental health [2005–2021; (A)], those warning on the critical status of the Mental Health of International Students during the COVID-19 Pandemic [2020–2021; (B)] and update of emerging literature (2022–2023) on this issue (C).

Authors	Scenario
A. Logotherapy and mental health (2005–2021)	
Schulenberg et al. (17)	Logotherapy for clinical practice.
Kang et al. (29)	The effects of logotherapy on meaning in life and quality of life of late adolescents with terminal cancer.
Smith (30)	Innovative applications of logotherapy for military-related PTSD.
Kang et al. (31)	Effects of logotherapy on life respect, meaning of life, and depression of older school-age children.
Mosalanejad and Khodabakshi (32)	Looking at infertility treatment through the lens of the meaning of life: The effect of group logotherapy on psychological distress in infertile women.
Delaviri et al. (33)	Logotherapy effect on anxiety and depression in mothers of children with cancer.
Jahanpour et al. (34)	The study of group logotherapy effectiveness on self-esteem, happiness, and social sufficiency in Tehranian girl teenagers.
Mohabbat-Bahar et al. (35)	Efficacy of group logotherapy on decreasing anxiety in women with breast cancer.
Mohammadi et al. (36)	Effectiveness of logotherapy in hope of life in the women depression.
Robatmili et al. (37)	The effect of group logotherapy on meaning in life and depression levels of Iranian students.
Soetrisno et al. (38)	The effect of logotherapy on the expressions of cortisol, HSP70, Beck Depression Inventory (BDI), and pain scales in advanced cervical cancer patients.
Baumel and Constantino (39)	Implementing logotherapy in its second half-century: Incorporating existential considerations into personalized treatment of adolescent depression.
Mortell (40)	Logotherapy to mitigate the harmful psychological effects of current events: A tool for nurses.
Bahar et al. (41)	Effectiveness of logotherapy on death anxiety, hope, depression, and proper use of glucose control drugs in diabetic patients with depression.
Kim and Choi (42)	The efficacy of group logotherapy on community-dwelling older adults with depressive symptoms: A mixed methods study.
Liu et al. (43)	Effects of logotherapy-based mindfulness intervention on internet addiction among adolescents during the COVID-19 pandemic.
Sun et al. (44)	The effects of logotherapy on distress, depression and demoralization in breast cancer and gynecologic cancer patients, a preliminary study.
B. Mental health of international students during the COVID-19 pandemic	
Lai et al. (45)	Mental health impacts of the COVID-19 pandemic on international university students, related stressors, and coping strategies.
Alam et al. (46)	Psychological outcomes and associated factors among the international students living in China during the COVID-19 Pandemic.
Kim and Kim (47)	Factors associated with mental health among international students during the COVID-19 pandemic in South Korea.
Lai et al. (48)	A phenomenological study on the positive and negative experiences of Chinese international university students from Hong Kong studying in the U.K. and U.S. in the early stage of the COVID-19 pandemic.
Matos Fialho et al. (49)	Perceptions of study conditions and depressive symptoms during the COVID-19 pandemic among university students in Germany: Results of the international COVID-19 student well-being study.
Negash et al. (50)	Worsened financial situation during the COVID-19 pandemic was associated with depressive symptomatology among university students in Germany: Results of the COVID-19 international student well-being study.
Song et al. (10)	COVID-19-related traumatic effects and psychological reactions among international students.
Van de Velde et al. (51)	The COVID-19 international student well-being study.
Yuan et al. (52)	Prevalence and predictors of anxiety and depressive symptoms among international medical students in China during COVID-19 pandemic.
C. Update on emerging literature on the critical status of the mental health of international students during the COVID-19 pandemic (2022–2023)	
Iftikhar et al. (8)	Prevalence of mental health problems among stranded international students during the COVID-19 pandemic
Maleku et al. (9)	Discrimination and mental health among international students in the US during the COVID-19 pandemic
Ke et al. (53)	The mental health of international university students from China during the COVID-19 pandemic and the protective effect of social support: A longitudinal study
Antwi et al. (54)	COVID-19 Pandemic and International Students’ Mental Health in China: Age, Gender, Chronic Health Condition, and Having an Infected Relative as Risk Factors
Jagroop-Dearing et al. (55)	Perceived Stress and Wellbeing among International Health Students Who Were Essential Frontline Workers during the COVID-19 Lockdown in New Zealand
Russell et al. (56)	Changes in Mental Health Across the COVID-19 Pandemic for Local and International University Students in Australia: A Cohort Study
Mihrshahi et al. (57)	Higher Prevalence of Food Insecurity and Psychological Distress among International University Students during the COVID-19 Pandemic: An Australian Perspective
Park and Shimada (58)	The impact of changing nonimmigrant visa policies on international students’ psychological adjustment and well-being in the United States during the COVID-19 pandemic: a qualitative study
Dong et al. (59)	“I Have a Wish”: Anti-Asian Racism and Facing Challenges Amid the COVID-19 Pandemic Among Asian International Graduate Students
Zhang et al. (60)	International student stressors and mental health during the COVID-19 pandemic: a qualitative study
Park and Shimada (58)	Mental health of international students in the United States during the COVID-19 pandemic and its relevant political climate: A descriptive cross-sectional study
Jamshaid et al. (61)	Pre- and Post-Pandemic (COVID-19) Mental Health of International Students: Data from a Longitudinal Study
Xiong et al. (62)	International students’ perceived discrimination and psychological distress during the COVID-19 pandemic
Lynch et al. (63)	international student trauma during COVID-19: Relationships among mental health, visa status, and institutional support
Dong et al. (64)	Relationships between racial discrimination, social isolation, and mental health among international Asian graduate students during the COVID-19 pandemic
Abukhalaf et al. (65)	Evaluating the mental health of international students in the U.S. during the COVID-19 outbreak: The case of University of Florida
Lu et al. (66)	Association of Covid-19 pandemic-related stress and depressive symptoms among international medical students
Yu et al. (67)	Mental health conditions of Chinese international students and associated predictors amidst the pandemic
Olatunji et al. (68)	COVID-19: Academic, Financial, and Mental Health Challenges Faced by International Students in the United States Due to the Pandemic
Gao et al. (69)	The experiences and impact on wellness among international students in the United States during the COVID-19 pandemic
Reid et al. (70)	COVID-19 stress, social support, and coping in international students during the COVID-19 pandemic: a moderated analysis on anxiety and depression
Um MY et al. (71)	Mask wearing and self-harming thoughts among international students in the United States during COVID-19: The moderating role of discrimination
Chen et al. (72)	Fear of COVID-19 and the career maturity of Chinese international high school students: The mediating effect of the intolerance of uncertainty
Collins et al. (73)	Urban green space interaction and wellbeing - investigating the experience of international students in Berlin during the first COVID-19 lockdown”
Lin et al. (74)	Prevalence and correlates of depression and anxiety among Chinese international students in US colleges during the COVID-19 pandemic: A cross-sectional study
Spatafora et al. (75)	Fear of Infection and Depressive Symptoms among German University Students during the COVID-19 Pandemic: Results of COVID-19 International Student Well-Being Study
Al-Oraibi et al. (76)	Exploring the Psychological Impacts of COVID-19 Social Restrictions on International University Students: A Qualitative Study
Okeke (77)	Compared to COVID, HIV Is Nothing: Exploring How Onshore East Asian and Sub-Saharan African International Students in Sydney Navigate COVID-19 versus BBVs/STIs Risk Spectrum
Rekenyi et al. (78)	The Effects and Differences of Social Support, Depression, and Vital Exhaustion during the COVID-19 Pandemic among International and Domestic University Students
Lu et al. (66)	Analysis of Influencing Factors of Psychological Intervention on International Students in China after COVID-19: Hainan Province, China
Yuan et al. (52)	Comorbid anxiety and depressive symptoms and the related factors among international medical students in China during COVID-19 pandemic: a cross-sectional study
Tan et al. (79)	Influencing Factors of International Students’ Anxiety Under Online Learning During the COVID-19 Pandemic: A Cross-Sectional Study of 1,090 Chinese International Students
Tran et al. (80)	From Academic Resilience to Academic Burnout among International University Students during the Post-COVID-19 New Normal: An Empirical Study in Taiwan
Andreatta et al. (81)	Context-Dependent Responses to the Spread of COVID-19 Among National and International Students During the First Lockdown: An Online Survey

### Design of the logotherapy intervention protocol

**Figure 1:**
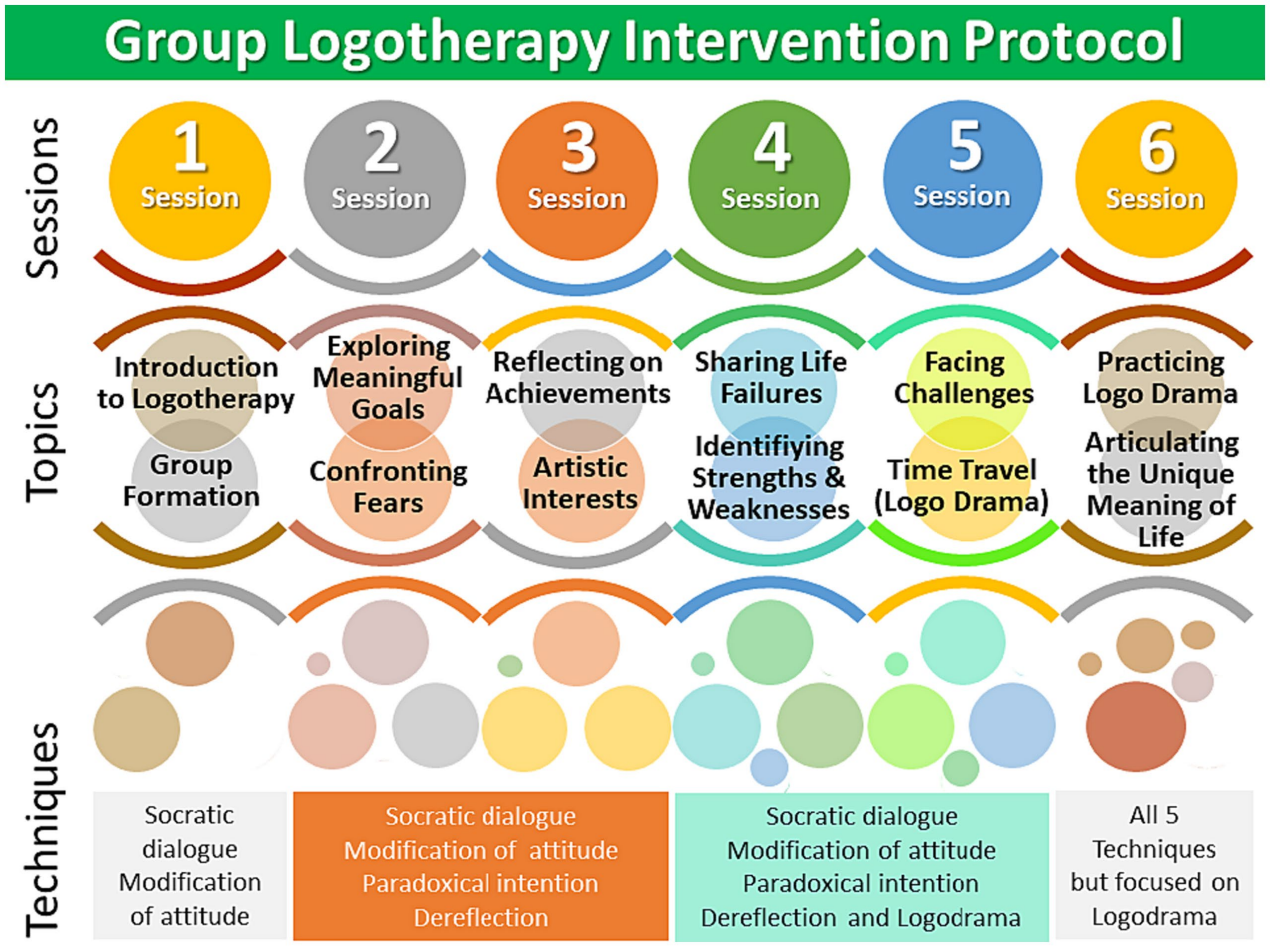
Designed group logotherapy intervention protocol: sessions, topics and techniques used.

The logotherapy intervention was designed as short-term closed group sessions, with each session lasting 90 min throughout 6 sessions. The control group also had the same number and duration of sessions; however, no specific psychological treatment was administered to this group (82). The logotherapy intervention, topics, and tools are described below and summarized in Figure 1.

### Session structure and objectives

The sessions were structured to facilitate engagement and meaningful participation of the participants. During Session One, essential introductory aspects of logotherapy were presented, including its founder, Victor Frankl, and its theoretical foundations. This set the stage for subsequent sessions, outlining a framework to navigate the complexities of participants’ mental well-being.

1. *Fundaments of Logotherapy* – In Session 1, participants were introduced to the fundamental concepts of logotherapy and the formation of the group, engaging in Socratic Dialog and Modification of Attitude techniques to set the stage for meaningful discussions.
2. *Existencial concerns* – During Session 2, participants explored meaningful life goals and confronted their fears, applying Socratic Dialog, Modification of Attitude, Paradoxical Intention, and Dereflection techniques to navigate these existential concerns.
3. *Introspection* – Session 3 involved reflecting on personal achievements and artistic interests, utilizing Socratic Dialog, Modification of Attitude, Paradoxical Intention, and Dereflection techniques to stimulate introspection.
4. *Self-awareness and growth* – In Session 4, participants shared their life failures and identified their strengths and weaknesses, applying Socratic Dialog, Modification of Attitude, Paradoxical Intention, and Dereflection techniques to promote self-awareness and growth.
5. *Empowering and facing challenges* – Session 5 focused on facing challenges and engaging in a time travel exercise (Logo Drama), incorporating Socratic Dialog, Modification of Attitude, Paradoxical Intention, Dereflection, and Logodrama techniques to empower participants and encourage continued daily relaxation practices.
6. *Meaning of life and conclusions* – During Session 6, participants practiced Logodrama and articulated the unique meaning of life, utilizing all five techniques with a particular focus on Logo Drama. The session provided a conclusion for all preceding sessions, effectively concluding the therapy series.

### Techniques and tools

Five logotherapeutic techniques were employed throughout the sessions: Socratic dialog, Paradoxical intention, Dereflection, modification of attitude, and Logodrama. These techniques were instrumental in encouraging active reflection, challenging assumptions, and fostering a shift in participants’ perspectives, essential for their mental health improvement.

1. *Socratic Dialog:* Engaging individuals in thought-provoking dialogs to facilitate self-reflection and a deeper understanding of their values and meaning (15).
2. *Modification of Attitude:* This technique involves altering one’s perspective and attitude toward an unavoidable situation, emphasizing the power of choice in interpreting the situation positively (13).
3. *Paradoxical Intention:* Encouraging individuals to confront their fears or anxieties often diminishes the fear’s hold over them (17).
4. *Dereflection:* Shifting focus from one’s problems by engaging in activities that direct attention away from the problem, aiding in achieving a healthier perspective (17).
5. *Logodrama:* Logodrama employs dramatic enactments or role-playing to explore and understand personal values, conflicts, and potential meanings in life (15).

### Tasks and reflection

Tasks assigned in each session were meticulously designed to encourage self-reflection and introspection, aiming to bring out meaningful insights from the participants. These tasks ranged from identifying personal values and fears to envisioning life goals within specific timeframes. Addressing these existential dimensions encouraged participants to confront their fears and anxieties, facilitating a transformative experience.

*Long-Term Impact –* The longitudinal nature of the sessions, reinforced by subsequent session tasks, emphasized continuity and practice, nurturing a sustained engagement with the principles of logotherapy. By envisioning and setting personal goals, acknowledging strengths and weaknesses, and engaging in therapeutic introspection, participants were better positioned to manage the challenges posed by the pandemic and enhance their mental well-being over time.

*Empowering the Participants –* The final session incorporated the powerful technique of logo drama, enabling participants to narrate their life journey, and emphasizing personal growth and resilience. This exercise aimed to empower the participants, highlighting their unique life narratives, thereby fostering a sense of purpose and meaning amidst adversities.

### Sample of participants

*Recruitment -* In response to our recruitment advertisement, over 130 individuals expressed interest in participating in the study. Following a thorough review process against the established inclusion and exclusion criteria, approximately 80 volunteers were selected to proceed to the clinical interview stage. The remaining 50 or so individuals were not selected for various reasons such as not meeting the specific research criteria, being outside the age range, using psychological medications, or undergoing other forms of psychotherapy.

*Dropouts and treatment adherence –* Adherence to the treatment protocol was a critical inclusion criterion for analysis. Consequently, only data from those who attended all six 90-min sessions and fully participated in the required exercises and homework was included. This process ensured that the treatment effects measured were based on complete participation, providing a clear and undiluted assessment of the intervention’s effectiveness.

*Final sample of participants* – Finally, this study involved 70 participants who suffered from moderate anxiety and depression according to their pre-tests. Three experimental sets (Set 1: December/ 2021–January/ 2022; Set 2: March–April/2022; Set 3: November–December/2022) were needed to achieve the total sample size. In each set, the participants were randomly divided into two groups, counterbalanced per sex/gender. The final composition was logotherapy group (*n* = 35) and the Control group (*n* = 35).

### Effectiveness of the logotherapy intervention protocol in Iranian international students

**Figure 2:**
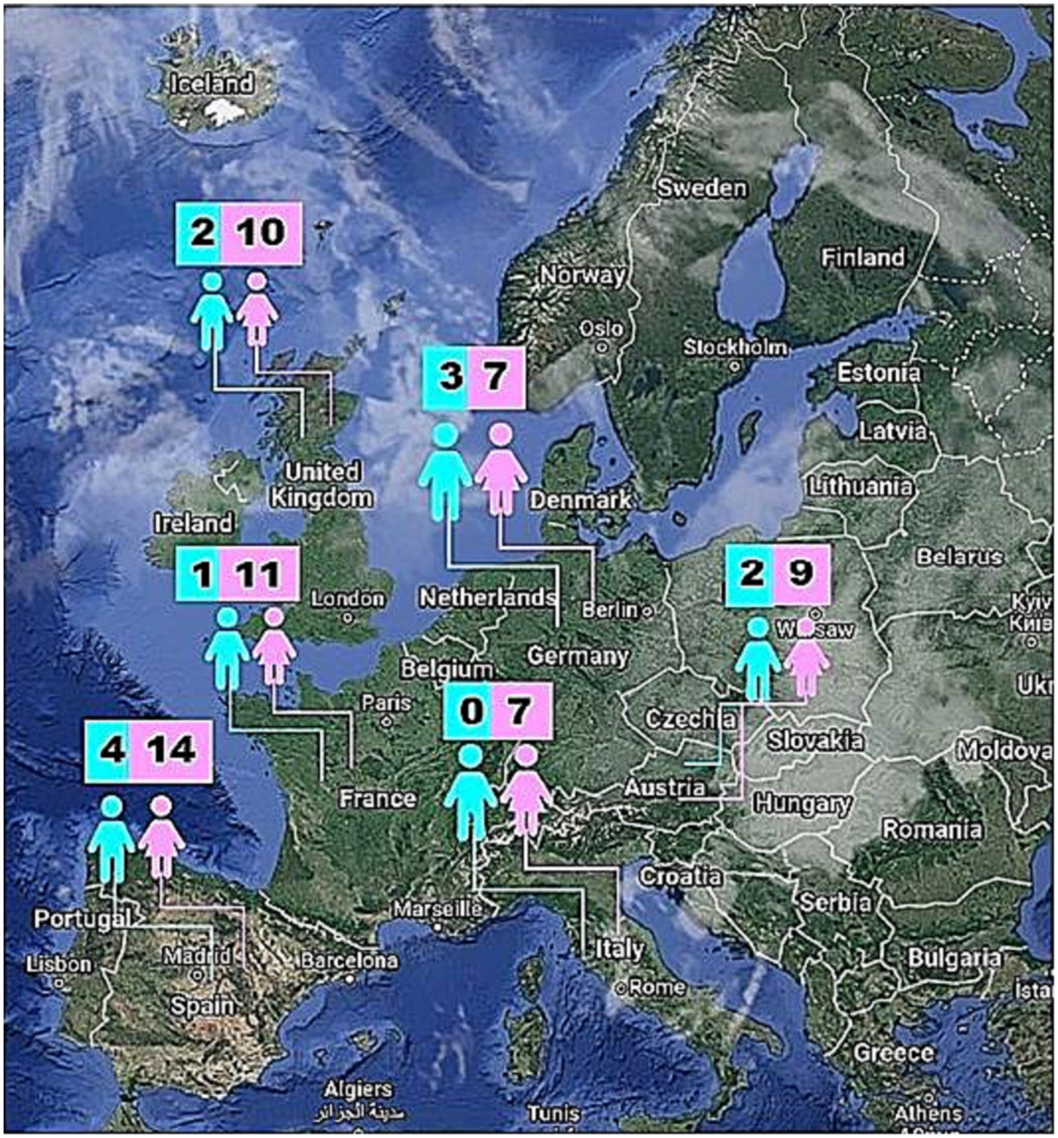
Geographical distribution per sex of Iranian international student participants in Europe during the COVID-19 pandemic.

Participants living in 6 different countries (Spain, UK, France, Austria, Germany and Italy) were randomly divided into the control (*n* = 35, 6 males and 29 females) and the logotherapy group (*n* = 35, 5 males and 30 females) balanced per sex. The age range for participants ranged from a minimum age of 20 to a maximum age of 35, showcasing a focused span of ages within the study. The mean scores for post-anxiety and post-depression were calculated for the overall sample and further stratified by group and per gender (Figure 2).

Multivariate analysis of covariance indicated a significant effect of ‘treatment group’ on anxiety and depression scores (*F* = 204.084, 2 dg, *p* < 0.001), with an eta squared coefficient (Pillai’s trace, η^2^ = 0.870) indicating that the observed difference accounted for 89% of the variance in the logotherapy intervention. In addition, the univariate analysis of covariance indicated that logotherapy significantly affected anxiety (*F* = 267.490, 1 dg, η^2^ = 0.812, *p* < 0.001) and depression (*F* = 208.810, 1 dg, η^2^ = 0.771, *p* < 0.001) scores in the logotherapy group when compared to the respective scores in the control group.

**Figure 3:**
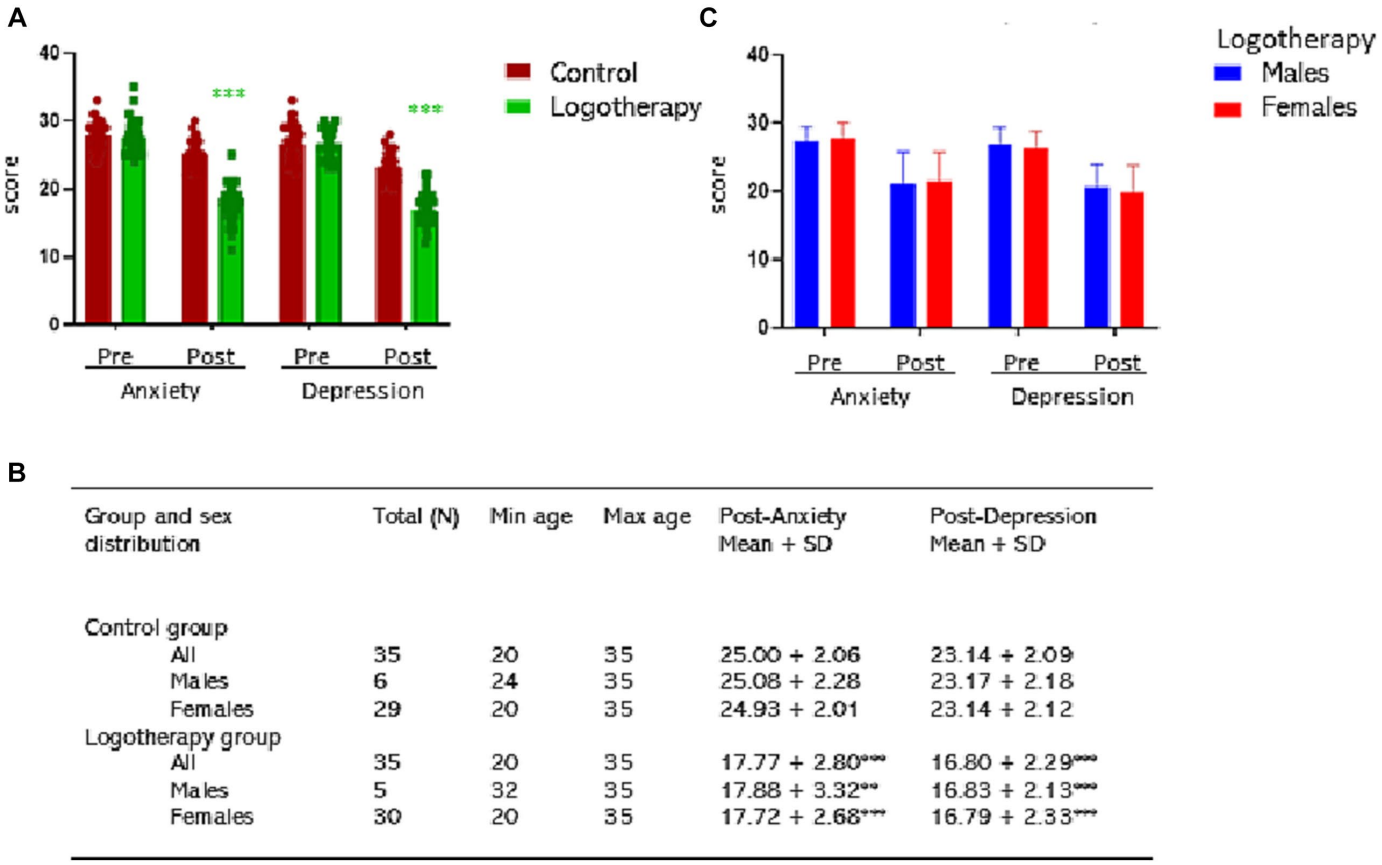
Pre- and post-test anxiety and depression scores. Results are expressed as individual values and/or mean + SD. **(A)** Control and logotherapy groups; **(B)** Post-test scores of **(A)** depicted per sex; **(C)** Pre- and post-test anxiety and depression scores in the logotherapy group per sex. Statistics: **p < 0.01, ***p < 0.001 vs. respective control group.

Figure 3A illustrates the anxiety and depression scores among control and logotherapy group participants before (Pre-test, both between groups differences were *n.s.*) and after (Post-test, Anxiety, *t* = 12.3048, 68 df, *p* < 0.0001 vs. control group; Depression, *t* = 12.0980, 68 dg, *p* < 0.0001 vs. control group) group sessions. Post-test results are also depicted per males and females in the table (Figure 3C, Anxiety, Males: *t* = 4.2610, 9 df, *p* = 0.0021 vs. control males; Females: *t* = 11.6594, 57 df, *p* < 0.0001 vs. control females; Depression, Males: *t* = 4.8520, 9 df, *p* = 0.0009 vs. control males; females: *t* = 10.9379, 57 df, *p* < 0.0001 vs. control females). No gender differences were found in the efficiency of logotherapy (Figure 3B).

## Discussion

### Logotherapy: an introduction to meaning-centered psychotherapy

Logotherapy, an existential psychotherapy approach developed by Viktor E. Frankl, is founded on the belief that the primary human drive is to find purpose and meaning in all circumstances (13, 14). The crux of logotherapy lies in the relentless pursuit of meaning, even in the face of suffering and adversity (15). The four key tenets of logotherapy are (1) Search for Meaning: Individuals are driven to seek meaning in life, in their actions, experiences, and relationships (13, 14); (2) Freedom of Will: Despite circumstances, individuals possess the freedom to choose their attitude toward situations and how they derive meaning from them (13); (3) Responsibility: logotherapy emphasizes taking responsibility for one’s life, choosing how to respond to situations, and thus ensuring a sense of purpose and meaning (13); (4) Suffering and Meaning: Suffering is seen as an opportunity to find meaning, to transform it into a triumph of the human spirit through the search for purpose (16).

### Logotherapy and improvement of mental health

Logotherapy has been widely applied in clinical practice to help individuals overcome mental health challenges. However, some critics argue that while logotherapy’s focus on meaning is valuable, it may oversimplify the complexities of mental health issues (83). The literature review presented a substantial body of evidence supporting the positive effects of logotherapy on different mental health outcomes. Here, we discuss the notable findings and trends observed in these studies:

The tailored application of logotherapy to address unique circumstances highlights its versatility and adaptability. The existential dimensions addressed by logotherapy resonate with individuals dealing with life-threatening illnesses and challenges, offering a sense of purpose and meaning. Thus, logotherapy has effectively addressed anxiety and depression across various existential scenarios such as mothers of children with cancer and individuals with advanced cancer (33, 37, 84); managing distress, demoralization, and hopelessness in cancer patients (44, 85); reducing existential loneliness and anxiety about death (85), enhancing hope of life (36), and improving self-esteem and happiness among teenagers (34). In other specific populations, such as diabetic patients with depression, logotherapy has effectively reduced death anxiety, increased hope, and improved medication compliance (41). The potential of logotherapy in enhancing spiritual well-being is also evident as shown in male cardiovascular patients were also reduced their anxiety (86). This indicates that logotherapy transcends the psychological domain and extends to the spiritual realm, promoting holistic well-being.

Logotherapy’s efficacy is not limited to physical health conditions; it extends to mental health challenges various demographics face. For instance, group logotherapy has benefited community-dwelling older adults with depressive symptoms Kim and Choi (42), adolescents struggling with internet addiction (43) or cyberbullied during the COVID-19 pandemic targeting depressive symptoms (87). These findings suggest the potential of logotherapy in addressing contemporary mental health issues.

Overall, the extensive body of research underscores the positive effects of logotherapy on mental health. From reducing anxiety and depression to enhancing existential well-being and spiritual dimensions, logotherapy stands as a promising psychotherapeutic approach for improving mental health outcomes across diverse populations.

### Mental health problems among international students during the COVID-19 pandemic

The COVID-19 pandemic has had a profound impact on global mental health, especially among international students. Recently, emergent studies are providing a comprehensive view of the mental health struggles experienced by international students during the pandemic. Notably, the prevalence of mental health problems, including anxiety and depression, among this demographic has been a significant concern. Studies such as those by Iftikhar et al. (8) and Kim and Choi (42) shed light on the prevalence of mental health problems, highlighting the need for targeted interventions. Additionally, factors exacerbating mental health issues among international students have been identified. Discrimination has emerged as a prominent factor affecting international students’ mental health during the pandemic, as evidenced by the study of Maleku et al. (9). Furthermore, the study by Antwi et al. (54) highlights how factors such as age, gender, chronic health conditions, and having an infected relative can contribute to mental health challenges. Moreover, the interplay between social support and mental health has been explored. The study by Ke et al. (53) underscores the protective effect of social support on international students’ mental health. Conversely, studies like Reid et al. (70) demonstrate that lack of social support can exacerbate anxiety and depression during the pandemic.

The experiences and effects of the pandemic and related stressors on international students vary across different contexts. Studies such as those by Um et al. (71) and Yuan et al. (88) provide insights into how discrimination, fear of infection, academic challenges, and social restrictions impact mental health outcomes. Additionally, some studies like Collins et al. (73) explore the role of environmental factors, such as access to green spaces, in mitigating stress among international students.

In summary, the mental health of international students during the COVID-19 pandemic is a multifaceted issue influenced by various factors including discrimination, social support, fear of infection, and academic challenges. Addressing these challenges requires tailored interventions considering the unique circumstances of international students, thus highlighting the importance of research in informing targeted mental health support.

### Efficacy of group logotherapy sessions enhancing the mental health of Iranian international students during the COVID-19 pandemic

The group logotherapy sessions conducted in this study aimed to address the mental health challenges faced by Iranian international students due to the secondary impact of the COVID-19 pandemic. The purpose was to mitigate moderate anxiety and depression, prevalent among this demographic sample, by employing the designed logotherapeutic intervention. In the results section and here, several key concepts of the study and intervention are dissected and discussed.

*Interpretation of Results –* The MANCOVA analysis revealed a significant effect of logotherapy on anxiety and depression, supported by an impressive η^2^ value of 0.89. This indicates a substantial enhancement in mental health among participants, affirming the potency of logotherapy as an intervention.

*Connection to Hypothesis –* The notable impact of logotherapy on anxiety and depression corroborates our initial hypothesis, emphasizing its effectiveness in reducing mental health challenges linked to the pandemic’s secondary impact. These findings underscore logotherapy’s potential as a valuable psychotherapeutic approach in addressing mental health issues during challenging times like the pandemic.

*Comparative Analysis –* Aligning with our initial hypothesis, which postulated logotherapy’s effectiveness in alleviating anxiety and depression exacerbated by the pandemic’s secondary impact, this study underscores the significance of logotherapy in the mental health domain. The empirical evidence presented here stands in harmony with existing literature, corroborating the positive influence of logotherapy on mental health, as demonstrated by Adhiya-Shah (89), Längle and Klaassen (90), Lewis (91), and Martínez and Flórez (92). Our study further adds to this body of evidence, emphasizing its efficacy within the context of Iranian international students during the pandemic.

*Control group* – The control group did not receive any specific psychological therapy. Despite the study’s single-blind design, where participants were unaware of their group assignment, we maintained the integrity of the control condition. In the first session, after participants were acquainted, the therapist engaged the group in a discussion about Victor Frankl’s life story, especially his experiences during the Holocaust. While the conversation initially centered on Frankl’s life, it gradually shifted to more general yet engaging topics. For example, we posed hypothetical scenarios to the participants, such as what choices they might make if they had one billion dollars. These discussions were designed to be thought-provoking and to foster group interaction without providing any therapeutic intervention.

It is important to note that these sessions were structured to control for participant engagement and therapist contact time without introducing therapeutic elements. This approach was taken to ensure that any differences observed between the control and treatment groups could be attributed to the logotherapy intervention itself rather than to nonspecific factors such as group cohesion or discussion on meaningful topics.

*Critical Mental Health Status of the International Students* – The comprehensive review of the literature on the mental health of international students since the beginning of the COVID-9 pandemic, when the current project was started, was corroborated by an important number of emerging studies that put efforts to provide scientific evidence of their critical status. As summarized, various aspects of the mental health of international students during the COVID-19 pandemic have been reported worldwide (8, 9, 53) (more recorded in Tables 1B,C).

*Gender Differences and Implications* Although, in our study, the analysis result of reducing anxiety and depression in both female and male were almost similar, in delving into the results of this study, it is essential to address the gender disparity among participants and its implications on mental health. Our research revealed a substantial representation of females (84%) in the study compared to males (16%) (33, 35). This aligns with existing research indicating a higher prevalence of mental health challenges, including anxiety and depression, among females. Notably, females were more inclined to engage in therapy groups and express themselves openly during both individual and group sessions (34, 87). The prevalence of females participating in the logotherapy group underscores the importance of tailoring interventions to address the specific mental health needs of this demographic.

*Age-Related Insights and logotherapy’s Efficacy* – Participants, aged 20–35, brought a range of life experiences to the group logotherapy sessions. Their varied perspectives, shaped by the shared challenge of the COVID-19 pandemic, enhanced group dynamics and supported a rich therapeutic dialog. This diversity proved beneficial, allowing participants to share and leverage coping strategies, which enriched the intervention’s effectiveness without being hindered by age differences. Such dynamics illustrate the adaptability of logotherapy across life stages and its potential to inform on how age-related factors contribute to therapy’s impact.

*Clinical Effectiveness and Participant Transformations –* The logotherapy sessions yielded outcomes that surpassed expectations. Participants reported significant personal revelations that aided in alleviating anxiety and depression, resonating with literature that documents similar therapeutic successes Kim and Choi (42). Clinically, these sessions fostered a sense of purpose and self-awareness among participants, empowering them to face life’s challenges more robustly. The process validated logotherapy’s effectiveness, particularly for our demographic of Iranian international students in Europe during the pandemic.

*Personal Growth and Meaning Reconstruction –* Participants’ accounts of personal transformation highlighted the profound impact of logotherapy. Many described a shift from existential despair to discovering personal significance and purpose, a finding consistent with the core principles of logotherapy. These narratives underscore the therapy’s power in catalyzing a redefinition of life’s meaning, even amidst adversity, providing strong clinical support for its use as detailed in existing literature. For example, one participant initially expressed a sense of nihilism, stating in the first session that “life is meaningless and not valuable.” However, by the end of the program, her viewpoint had shifted dramatically. She recognized that her initial belief was a reflection of her despair and reported that she had found profound and personal meanings in her life, declaring it to be valuable and purposeful. Similar sentiments were echoed by the majority of participants.

*Impact of Context and Online Sessions –* Furthermore, as this study involved Iranian international students dispersed across various European countries, it is crucial to acknowledge the impact of different contextual and country-specific scenarios on the effectiveness of logotherapy (33, 93). Despite the varied contexts, logotherapy consistently proved effective in alleviating mental health challenges among the participants. Particularly noteworthy was the utilization of online sessions for group therapy, overcoming physical barriers and enabling individuals to engage in therapy despite being alone in a foreign country (87, 93). This highlights the adaptability and accessibility of logotherapy, especially in the context of the COVID-19 pandemic.

*Online Group Therapy –* The group therapy sessions in this study were conducted online, a mode of intervention that has been gaining prominence in recent times (87). Online therapy has proven to be effective and accessible, overcoming geographical barriers and allowing individuals to participate in therapy sessions from the comfort of their own space (40). This online approach was especially relevant for our study, where participants were Iranian international students located across different European countries, emphasizing the significance and versatility of remote interventions.

*Strength of the current study* – The comparison with existing literature is essential in highlighting the novelty and importance of our research. While our study contributes to this body of research, it stands out with its unique characteristics: (1) While there are studies that explore mental health in international student populations, our research fills a gap by explicitly focusing on the unique experiences of Iranian international students during the COVID-19 pandemic. Our study specifically targets Iranian international students who are migrants or refugees in European countries, a population that faced distinct challenges during the pandemic with additional stressors due to being young immigrants/refugees. This scenario can be more significant for females (11). Therefore, inclusion of predominantly female participants in our study population is noteworthy, as it sheds light on the mental health challenges this specific demographic population faces. (2) The outcomes of the present study underscore the significance of logotherapy as a promising approach for enhancing psychological well-being in complex scenarios. While contributing to the growing literature on mental health interventions for international students during the pandemic, the present work designed, implemented and assessed the effectiveness of an online logotherapy intervention, providing a valuable avenue for addressing the mental health concerns of migrant and refugee students. (3) Additionally, our study incorporates a logotherapy intervention delivered through online platforms, a distinctive approach compared to previous studies. The use of online platforms allows for overcoming geographical barriers, isolation and loneliness and enhances accessibility to mental health interventions for this population. It is also important to note that, while in this study the interventions were offered for free, in any other case online sessions can help to reduce costs. To the best of our knowledge, few previous studies have explored logotherapy specifically in an online context for international student populations. This integration of logotherapy principles and techniques via online platforms provides a novel and innovative approach to addressing mental health concerns in this student population during challenging circumstances under a discreet access format.

*Replication of the Study –* To replicate the study and achieve comparable results, the group protocol requires an individual who is both a trained psychotherapist and a trained Logotherapist. The delivery of the logotherapy intervention requires not just familiarity with its principles but also the ability to apply them therapeutically, which necessitates specific training in logotherapy techniques. In addition, the psychotherapist’s broader clinical skills are essential for managing group dynamics and addressing any clinical issues that may arise during the sessions.

*Limitations –* Acknowledging limitations is crucial for comprehensively understanding the study’s scope. While appropriate for this study, the sample size may somewhat constrain the generalization of the results to a broader population. Additionally, variations in cultural contexts among international students could introduce potential biases and influence the study’s outcomes. These limitations highlight the need for cautious interpretation and encourage future research to address potential biases and contextual factors.

*Practical applications –* For these results to be translated into real-world strategies and interventions to support the mental well-being of Iranian international students in Europe, identifying actors, niches and resources is essential. We consider that first actors should be universities, as the educational but also social niche of these subjects, and the ones with immediate and stronger capacity to help them counteract stigma and loneliness. Conversely, the university community can be seen as a precious change source. Some universities already have programs to care for the mental health of (any) students, providing external (but also internal) professional support, and they have experienced a worrisome increase in demand in this new COVID-19 pandemic era. Specific programs for their international students, mostly immigrants/refugees, will likely be driven by their associated NGOs in collaboration with National CAR (Committees for Refugee Assistance). For instance, Universitats Refugi (Refugee program) from Fundació Autònoma Solidaria, Universitat Autònoma de Barcelona, and Catalonian CAR (94) have specific ‘welcome-training-awareness-network’ social programs aimed to boost the university’s capacity to offer assistance and uphold the rights of migrants/refugees. Similarly, at the international level, UNICA (Institutional Network of Universities from the Capitals of Europe) also has the ‘Academic Refuge’ (95), a strategic partnership to promote core academic values and welcome refugees and threatened academics to European campuses, with granted financial support from the European Commission under the Erasmus+ program managed by Norwegian National Agency. However, specific mental health programs should complement social programs if they want to address the current gap that exists between academic/social and clinical support.

*Future directions –* To build on this study, future research could explore tailored applications of logotherapy in various cultural contexts, incorporating insights from the referenced literature. As suggested by Zhang et al. (60), investigating the long-term effects and sustainability of logotherapy interventions is essential for a comprehensive understanding of its lasting impact on mental health. Moreover, comparative studies could assess the effectiveness of logotherapy in diverse populations, as proposed by Adhiya-Shah (89), to further validate its potential as a universal mental health intervention.

## Conclusion

In conclusion, in the present study, we designed and demonstrated the efficacy of an online logotherapy intervention reducing the anxiety and depression of Iranian international students who are migrants or refugees in Europe during the COVID-19 pandemic.

Our study contributes to the existing literature on the mental health of international students in this challenging period and unveils two key findings: (1) The benefits of online group sessions, highlighting the effectiveness of delivering interventions despite geographical distance, (2) The relevance of logotherapy in effectively reducing depression and anxiety in the unique and challenging contexts where psychological resources and cultural competencies are limited. Through targeted interventions and structured sessions incorporating logotherapy techniques, such as paradoxical intention and modification of attitude, participants were encouraged to reflect, set goals, and envision a meaningful life. The sessions fostered introspection, self-awareness, and empowerment in participants, contributing to ameliorating moderate depression and anxiety prevalent within this population.

The findings of the present research advocate for integrating logotherapy into mental health interventions, offering a promising avenue for enhancing the well-being of individuals grappling with psychological challenges.

## Data availability statement

The raw data supporting the conclusions of this article will be made available by the authors, without undue reservation.

## Ethics statement

The studies involving humans were approved by CEEAH Comissió Ètica Experimentació Animal i Humana, Universitat Autònoma de Barcelona. The studies were conducted in accordance with the local legislation and institutional requirements. Written informed consent for participation in this study was provided by the participants’ legal guardians/next of kin.

## Author contributions

SR: Conceptualization, Data curation, Formal analysis, Investigation, Methodology, Writing – original draft, Writing – review & editing. LG-L: Conceptualization, Funding acquisition, Methodology, Project administration, Resources, Supervision, Validation, Writing – review & editing.
